# FieldDino: Rapid In‐Field Stomatal Anatomy and Physiology Phenotyping

**DOI:** 10.1111/pce.15639

**Published:** 2025-05-27

**Authors:** Edward Chaplin, Guy Coleman, Andrew Merchant, William Salter

**Affiliations:** ^1^ School of Life and Environmental Sciences, Sydney Institute of Agriculture The University of Sydney Sydney New South Wales Australia; ^2^ Department of Plant and Environmental Sciences, Faculty of Science University of Copenhagen Taastrup Denmark; ^3^ The Australian Plant Phenomics Network The University of Sydney Narrabri New South Wales Australia

**Keywords:** abiotic stress, handheld microscope, high‐throughput, machine learning, phenotyping, plant breeding, stomata, stomatal anatomy, stomatal conductance, wheat

## Abstract

Stomatal anatomy and physiology define CO_2_ availability for photosynthesis and regulate plant water use. Despite being key drivers of yield and dynamic responsiveness to abiotic stresses, conventional measurement techniques of stomatal traits are laborious and slow, limiting adoption in plant breeding. Advances in instrumentation and data analyses present an opportunity to screen stomatal traits at scales relevant to plant breeding. We present a high‐throughput robust field‐based phenotyping approach, FieldDino, for screening stomatal physiology and anatomy. The method allows measurements to be collected in < 15 s and consists of: (1) stomatal conductance measurements using a handheld porometer; (2) in situ collection of epidermal images with a digital microscope, 3D‐printed leaf clip and Python‐based app; and (3) automated deep‐learning analysis of stomatal features. The YOLOv8‐M model trained on images collected in the field achieved strong performance metrics with an mA*p*@0.5 of 97.1% for stomatal detection. When validated in large field trials of 200 wheat genotypes under two irrigation treatments, FieldDino captured wide diversity in stomatal traits. FieldDino enables stomatal data collection and analysis at unprecedented scales in the field. This will advance research on stomatal biology and accelerate the incorporation of stomatal traits into plant breeding programs for resilience to abiotic stress.

## Summary statement

FieldDino enables high‐throughput phenotyping of stomatal physiology and anatomy traits under field conditions, advancing our understanding of stomatal function and responses to abiotic stress. It presents an opportunity for incorporation of stomatal traits into crop improvement programs.

**Abbreviations**
*a'*
: Mean maximum stomatal pore area which is calculated assuming all stomatal pores are elliptical with the pore length equal to the major axis and the minor axis equal to half the pore length (υm^2^)
CAIGE: CIMMYT Australia ICARDA Germplasm Evaluation
CIMMYT: International Maize and Wheat Improvement Center
CO_2_
: Carbon Dioxide
COCO: Common Objects in Context
*d*
: Diffusivity of water in are at 22°C (m^2^ s^‐1^)
EDPIE: Elite Diversity International Experiment
ESWYT: Elite Selection Wheat Yield Trial
F1: Harmonic mean of precision and recall
FLOPs: Floating Point Operations per Second
*g*_*leaf, min*_
: Minimum leaf stomatal conductance
*g*_*s*_
: Stomatal conductance
*g*_*smax*_
: Maximum anatomical stomatal conductance
*g*_*sop*_
: Operating rate of stomatal conductance
*g*_*s*op_/*g*_*smax*_
: Stomatal conductance operating efficiency
H_2_O: Water
HTWYT: High Temperature Wheat Yield Trials
ICARDA: International Centre for Agricultural Research in the Dry Areas
IoU: Intersection over Union
IRGA: Infrared gas analysers
*l*
: Pore depth which is equal to the guard cell width at the centre of the stomata based on half the guard cell length (υm)
mAP: Mean average precision
Params: Parameters
ΦPSII: Quantum yield of electron transfer at PSII
PSII: Photosystem II
SAI: Stomatal Area Index
SATYN: Stress Adapted Trait Yield Nursery
SAWYT: Semi‐Arid Wheat Yield Trial
SD: Stomatal density (stomata m^‐2^)
*v*
: Molar volume of air at 22°C (m^3^ mol^‐1^)
YOLO: You Only Look Once

## 1 Background

Stomata control the exchange of gases between the intercellular air spaces of the leaf and the atmosphere. Despite links between stomata, photosynthesis and abiotic stress tolerance, stomatal traits have rarely been adopted as targets in plant breeding due to constraints in field measurement at appropriate scales. Significant yield gains in the second half of the 20th century, largely associated with increased harvest index (Porker et al. 2020), have dwindled recently as realised gains near the theoretical limit in elite populations (Furbank et al. 2020). Consequently, the focus of breeders has shifted to radiation‐use‐efficiency via increased photosynthetic capacity and efficiency (Parry et al. 2011), in which stomatal traits play a key role (Roche 2015). There is significant genotypic variation and heritability for stomatal conductance amongst wheat germplasm (Rebetzke et al. 2013; Faralli et al. 2024). Development of robust, high‐throughput screening approaches could allow us to translate these desirable traits into targets for plant breeding programs.

Combining measures of stomatal physiology and anatomy in phenotyping provides insights into plant function for trait development and modelling. The anatomy of stomatal cells, largely fixed after leaf expansion (Mano et al. 2023), influences stomatal area index (SAI) and maximum stomatal conductance (*g*_smax_) (Franks et al. 2009). Under optimal conditions, lower *g*_smax_ could limit potential CO_2_ assimilation (Wullschleger et al. 2002; Harrison et al. 2020), while under stress, a greater *g*_smax_ could lead to excessive water loss (Doheny‐Adams et al. 2012). High stomatal density and reduced stomatal size have been identified to be advantageous under drought, increasing *g*_*smax*_ to facilitate rapid gas exchange when water is readily available without causing a rise in the minimum leaf conductance, *g*_*leaf, min*_, which would be of detriment to drought tolerance (Ochoa et al. 2024).

Additionally, stomatal opening and closure are responsive, regulating instantaneous CO_2_ uptake and water loss relative to changes in the environment (Kollist et al. 2014; Merilo et al. 2014; Nunes et al. 2022). Typically, the operating stomatal conductance (*g*_*sop*_) is significantly lower than *g*_smax_ (Fanourakis et al. 2015; McElwain et al. 2016; Ouyang et al. 2017). However, *g*_s*op*_ and *g*_smax_ are positively correlated (Franks et al. 2009; McElwain et al. 2016). Combining measurements of stomatal physiology and anatomy allows calculation of stomatal conductance efficiency (*g*_*sop*_ */g*_*smax*_), a useful metric for assessing gas exchange efficiency and drought tolerance (Bertolino et al. 2019; Busch et al. 2024). This underscores the need to develop high throughput, field‐phenotyping methods that can effectively capture both physiology and anatomy data at field scales.

Until recently, measurement of stomatal conductance (*g*_*s*_) has been either (1) fast but lacking in precision with porometry or (2) precise but slow with gas exchange (Bell and Squire 1981; Monteith et al. 1988; Toro et al. 2019). Porometers are fast and field‐portable but vary in accuracy and precision with factors including environmental conditions (McDermitt 1990; Turner 1991; Fanourakis et al. 2016). By contrast, infrared gas analysers (IRGA) provide more accurate measurements (Toro et al. 2019), substantially contributing to our understanding of stomatal physiology (Guizani et al. 2023; Li et al. 2023; Wang et al. 2024c). IRGA's however are not well suited for high‐throughput phenotyping studies with slow measurement times (Taylor and Long 2017) and significant capital cost. Recently, handheld porometers with improved accuracy under fluctuating environmental conditions, such as the LI‐COR LI‐600 porometer/fluorometer, have enabled the rapid establishment of large datasets of *g*_*s*_ in wheat (McAusland et al. 2023) and other species (Bheemanahalli et al. 2022; Fenstemaker et al. 2022; Caine et al. 2023).

Similarly, image capture of stomatal anatomy has faced similar throughput limitations. Traditional approaches such as the nail polish ‘peel’ technique (Kapadiya et al. 2017; Wall et al. 2022, 2023) are time‐consuming and prone to contamination with artifacts (Millstead et al. 2020). Recently, handheld digital microscopes have emerged as an alternative for in situ epidermal imaging, offering faster and more reliable data collection across different species (Sun et al. 2021, 2023; Pathoumthong et al. 2023). With further optimisation, such tools can facilitate more extensive anatomical data collection under field conditions. Analysis of thousands of epidermal images to extract traits like stomatal density, size and shape has historically been a labour‐intensive and error‐prone process (Bertolino et al. 2019; Casado‐García et al. 2020) using manual methods with little automation such as ImageJ software (Schindelin et al. 2012). Recent advances in deep learning and computer vision allow highly accurate and automatic extraction of stomatal features in anatomical images of wheat (Gibbs et al. 2021; Sun et al. 2021, 2023; Pathoumthong et al. 2023) and other species (Meeus et al. 2020; Ren et al. 2021; Zhang et al. 2022; Wang et al. 2024b, 2024a). Using deep learning for stomatal annotation and measurement has significant potential to streamline stomatal phenotyping. Yet studies have largely been limited to a handful of genotypes grown under glasshouse conditions. Broadening model training datasets to encompass diverse plant material imaged in the field would likely improve the utility of model predictions for genotype selection in plant breeding operations.

Given the advances in porometry and microscopy, it is now feasible to screen for stomatal physiological and anatomical traits at scales relevant for plant breeding under field conditions. This study presents a three‐stage, high‐throughput phenotyping approach for stomatal conductance and stomatal anatomy traits: (1) handheld in situ porometer measurements of *g*_*s*_ and chlorophyll fluorescence, (2) streamlined in situ collection of anatomical images using a handheld microscope with custom microscope mount and software app, and (3) deep learning‐based automated image analysis. We validate this method in large wheat field trials containing 200 diverse genotypes and two irrigation treatments, offering a pathway to increase throughput and provide deeper insight into the roles of stomata in the future of crop improvement.

## 2 Methods

### 2.1 Plant Material

Field plots were established on 24th May 2023 at the University of Sydney I.A. Watson Grains Research Centre in Narrabri, NSW Australia (30.2743° S, 149.8093° E) with two water availability treatments; rainfed and irrigated. 200 genotypes of wheat (*Triticum aestivum* L.) were sown as part of a broader study focused on drought tolerance. Three field replicate plots of each genotype were sown per treatment arranged in randomised blocks. Plants were sown in 12 m^2^ plots (2 × 6 m) at a planting density of 100 plants m^−2^ with five planting rows. More detail on the germplasm is available in Supplementary Information; Appendix 1.

The field site contains predominantly black vertosol cracking clays with high water retention. Rainfed plants received 102.4 mm of rainfall and irrigated plants received 197.4 mm of total moisture (rainfall + irrigation) during the growing season. Fertiliser application was consistent across treatments (urea [46% N] 100 kg ha^−1^ and Cotton Sustain [5% N, 10% P, 21% K, 1% Z] 80 kg ha^−1^ pre‐planting). The trial was harvested on 26th October 2023.

### 2.2 Field Measurements

For measurements of stomatal physiology and anatomy, one plant was sampled per plot (*n* = 3 per genotype per treatment). Fully expanded flag leaves were measured at anthesis (Zadok/BBCH stage 48–68) and selected for measurement using a systematic randomised sampling technique, with representative plants of each plot selected from the middle three planting rows at least 0.5 m from the end of each plot. Data was collected on consecutive days where possible and all measurements were taken between 09:30 and 15:00 to minimise diurnal effects on rates of photosynthesis and stomatal opening/closure. For all leaves sampled, both the upper (adaxial) and lower (abaxial) leaf surfaces were measured.

#### 2.2.1 Stomatal Conductance

Stomatal conductance and chlorophyll fluorescence of the abaxial and adaxial leaf surfaces were measured using a LI‐COR LI‐600 porometer/fluorometer (LI‐COR Inc., Nebraska, USA) with the following settings, flow rate: 150 µmol s^−1^; flash intensity: 7,000 µmol m^−2^ s^−1^; phase length: 300 ms; ramp amount: 25%; leaf absorptance: 0.8; fraction abs PSII: 0.5; actinic modulation rate: 500 Hz; and integrated modulation intensity: 6.67 µmol m^−2^ s^−1^. The stability was set to the ‘medium’ preset to provide a balance between speed and accuracy. The porometer sensor head was ‘matched’ in the field at the start of the day with an empty chamber and automatically underwent ‘matching’ after every ten measurements. Measurements were timestamped and custom remarks were added to each log for ease of data processing and organisation.

Abaxial and adaxial measurements were made by placing the sensor head leaf clip on the middle portion of the leaf blade of the main tiller's flag leaf which was fully sunlit. The leaf was positioned off‐centre in the sensor head to avoid clamping the main vein of the leaf. Total stomatal conductance was calculated by combining the conductance recordings of abaxial and adaxial surfaces.

#### 2.2.2 Stomatal Anatomy Image Capture

Following sampling for stomatal conductance, the same leaf was excised from the plant for immediate in‐field microscopy imaging of the abaxial and adaxial leaf surfaces using a 200x magnification handheld USB microscope (Dino‐Lite 5MP, AM7515MT2A; Dino‐Lite, AnMo Electronics Corporation, Taiwan). The 200x magnification was chosen to provide a good balance between sample size for stomatal density and resolution for stomatal size measurements. The microscope was configured with LED brightness=1; LED axial light=0; and resolution=2592 ×1944 pixels. While our method is inherently non‐destructive and does not require leaf excision for imaging, in this experiment, leaves were excised for ease of imaging given tissue samples were also collected for nutrient analysis for an adjacent study.

**Figure 1:**
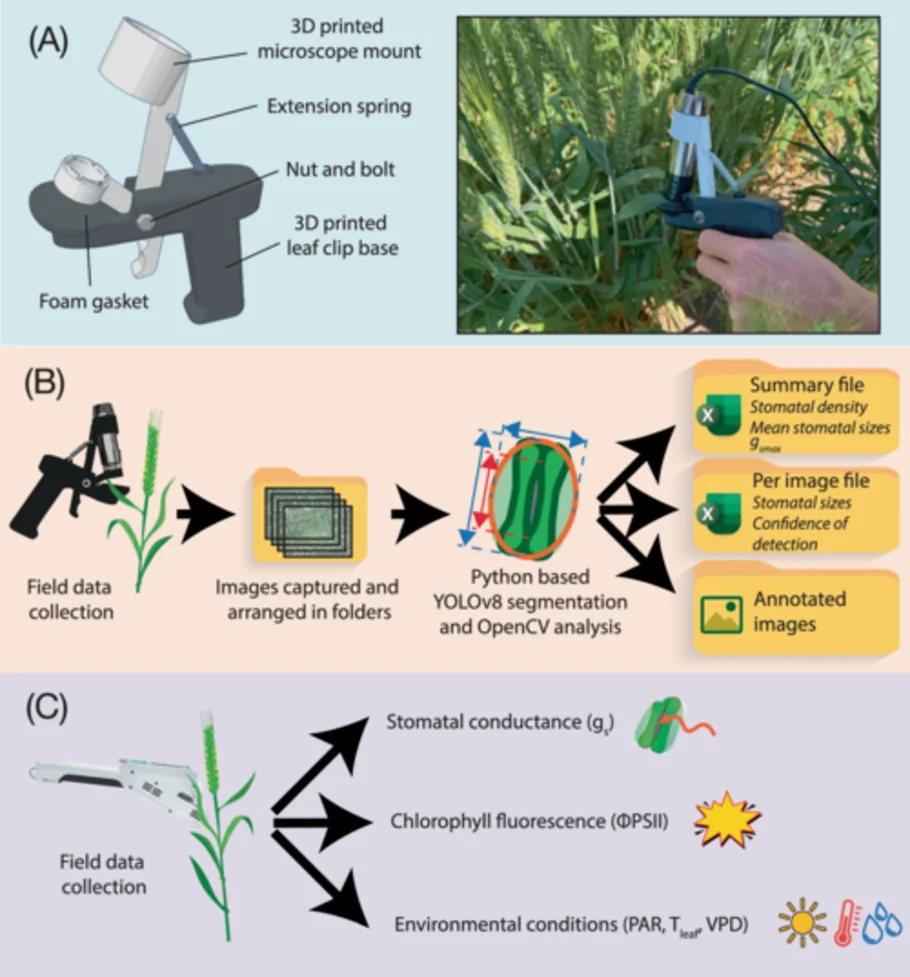
Schematic diagram of the presented stomatal phenotyping method. (A) the 3D printed leaf clip, it's assembly and in the photo, how the microscope is secured in the leaf clip. (B) and (C) highlight the traits measured by the handheld microscope and porometer, respectively.

The reliability of in‐field microscope focusing was improved with a custom 3D‐printed leaf clip with a smaller aperture than the original microscope cap. Designed in Tinkercad (Autodesk Inc., Mill Valley, CA, USA), STL files are provided in the FieldDino GitHub repository and can be customised for other Dino‐Lite microscopes. The leaf clip was printed in two parts: handle/base and trigger/microscope mount using black and white PETG filament, respectively (Figure 1), with the white filament also acting as a diffuser for the microscope LED lights. An M5 nut and bolt secured the two parts of the leaf clip, while a small piece of gasket foam and 38.1 mm extension spring provided a spring‐open mechanism. Black electrical tape was wrapped around the clear part of the microscope shaft to block external light and provide consistent lighting conditions for imaging. The microscope was connected by USB cable to a laptop computer. The adaxial and abaxial surface of each leaf was imaged by clamping the leaf into the leaf clip and focusing by adjusting pressure on the trigger. Images were automatically saved to the local hard drive in the order they were captured using DinoXcope software (v2.4.1; Dino‐Lite, AnMo Electronics Corporation, Taiwan) in PNG format.

#### 2.2.3 FieldDino: In‐Field Microscopy App

**Figure 2:**
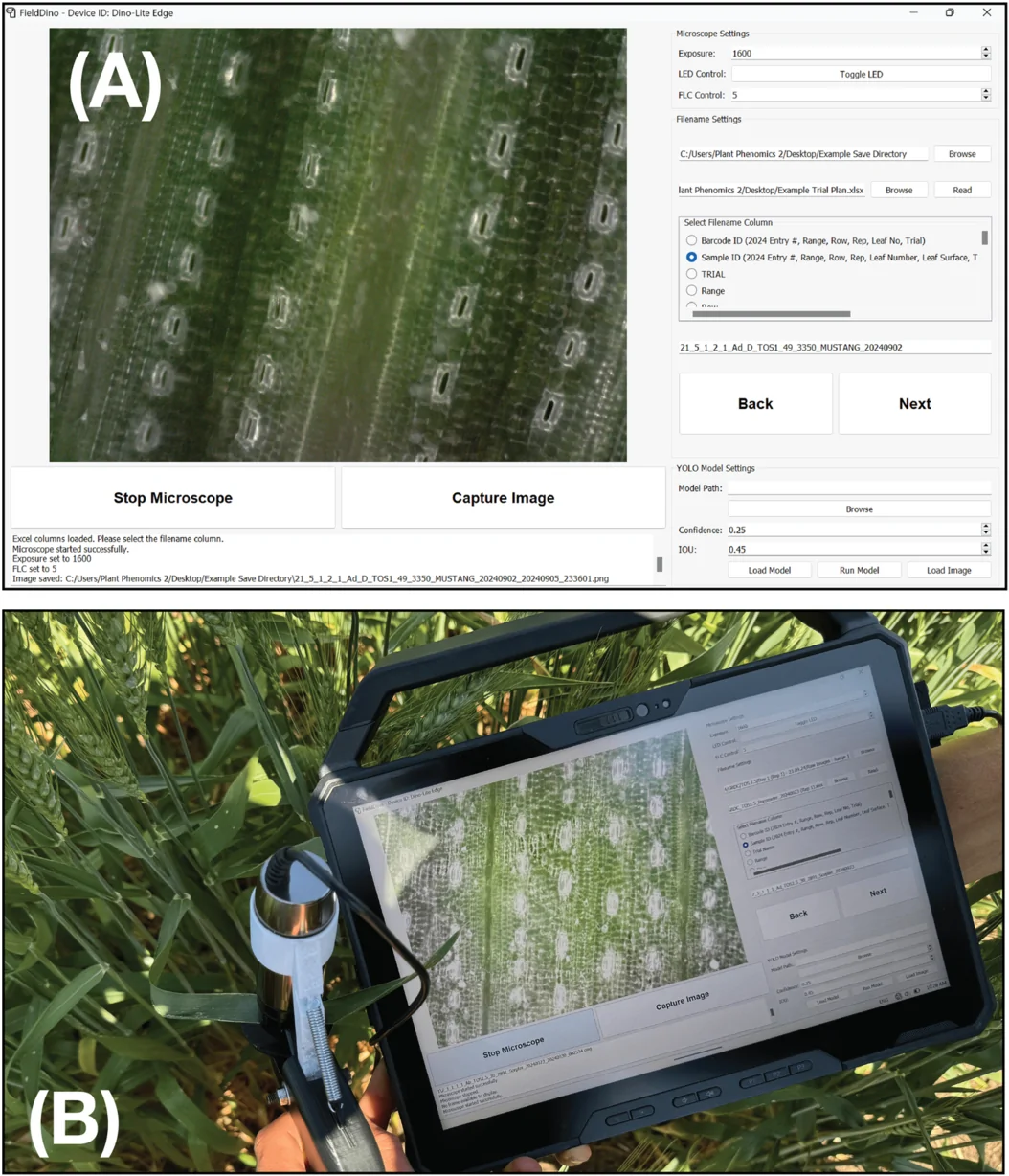
Image capture software. (A) FieldDino custom‐designed Python‐based app optimised for in‐field microscopy. Functionality includes image naming from excel file, a clear video feed with adjustable microscope parameters, easy to use buttons for image capture and ability to run real‐time stomatal detection algorithms. (B) handheld digital microscope, custom‐made microscope leaf clip and FieldDino app being used in the field for image capture. [Color figure can be viewed at wileyonlinelibrary.com]

Following the 2023 field campaign, we developed a custom Python based image capture app optimised for in‐field microscopy (Figure 2). The FieldDino app has been tested in subsequent field trials (data not shown) and incorporates several improvements over the Dino‐Lite software.

Built with Python and a PyQt5 graphical user interface, FieldDino reads image file names from a trial spreadsheet, streamlining image naming according to sampling design. Camera parameters are adjustable and recordable in a metadata text file. The app also supports real‐time deep learning stomata segmentation using Ultralytics and PyTorch libraries. Installation and guidance for the app are available on the FieldDino GitHub repository: https://github.com/williamtsalter/FieldDinoMicroscopy.

### 2.3 Data Analysis

#### 2.3.1 Image Annotation and Model Training

518 images (including samples from all 200 genotypes) collected in a single day were used for algorithm development. Stomata were annotated via instance segmentation using Roboflow (Roboflow Inc., Iowa, USA). YOLOv8 models of different sizes were trained and compared based on performance metrics with YOLOv8‐M ultimately chosen for further stomatal analyses (Table 1). More details of model development are provided in Supplementary Information; Appendix 2, Figures S1 & S2.

**Table 1:** Performance metrics used to compare different YOLOv8‐seg algorithms. Metrics used were: Parameters (Params), Floating Point Operations per Second (FLOPs), Precision (Mask), Recall (Mask), F1 Score (Harmonic mean of Precision and Recall), mA*p*@0.5 (mean average precision at Intersection over Union = 0.5) and mAP@[0.5:0.95] (mean average precision at Intersection over Union from 0.5 to 0.95). Based on these parameters, the YOLOv8 M model was chosen due to strong performance metrics balanced with a moderate model size.

Model algorithm	Size (Pixels)	Epochs	Params	FLOPs	Precision (M)	Recall (M)	F1 score	mA*p*@0.5	mAP@[0.5:0.95]
YOLOv8n	640	315	3.4	12.6	0.9630	0.9292	0.9458	0.9659	0.6401
YOLOv8s	640	115	11.8	42.6	0.9633	0.9239	0.9432	0.9659	0.6424
YOLOv8m	640	326	27.3	110.2	0.9651	0.9377	0.9512	0.9709	0.6592
YOLOv8l	640	456	46	220.5	0.9651	0.9360	0.9503	0.9700	0.6762
YOLOv8x	640	239	71.8	344.1	0.9665	0.9376	0.9518	0.9718	0.6682

#### 2.3.2 Measurement of Stomata

The 5th percentile of confidence intervals for use with model prediction was determined from model predictions on all 2,400 images. The confidence interval was then set to 75% and we ran a custom Python script (available on GitHub), utilising the Ultralytics package for model inference and OpenCV package for measurements, on all 2,400 images to quantify stomatal traits. The OpenCV script counts the number of stomata per image (stomata mm^−2^), the area of the stomatal cells (µm^2^) and then fits an ellipse around each stomata based on the contours of the polygon outputted by the deep learning model (Supplementary Information; Figure S3). Note, any stomata not fully within the image frame were excluded from analysis to ensure consistency across the data set and avoid measurement of portions of stomata at the edge of images. The fitted ellipses were used to estimate guard cell length (µm) and guard cell width (µm). A schematic of the approach and associated metrics is provided in Supplementary Information; Figure S1A.

#### 2.3.3 Guard Cell – Pore Length Relationship & Theoretical Anatomical Maximal Conductance

The anatomical maximum stomatal conductance assuming fully open pores (*g*_*smax*_) can be calculated from the stomatal dimensions and stomatal density and the assumption that pore length is approximately half guard cell length (Franks and Farquhar 2007; Franks and Beerling 2009). The following equation by Wall et al. (2022) can then be used to calculate *g*_*smax*_:

$$g_{\mathrm{smax}}=\frac{\left(d\times \mathrm{SD}\times a^{\prime }\right)}{v\times \left(l+\left(\frac{\pi }{2}\right)\times \sqrt{\frac{a^{\prime }}{\pi }}\right)}$$

Where: *d*=diffusivity of water in air at 22°C (m^2^ s^−1^); SD=stomatal density (stomata m^−2^); *v*=molar volume of air at 22°C (m^3^ mol^−1^); *l*=pore depth which is equal to the guard cell width at the centre of the stomata based on half the guard cell length (υm); *a*'=the mean maximum stomatal pore area which is calculated assuming all stomatal pores are elliptical with the pore length equal to the major axis and the minor axis equal to half the pore length (υm^2^).

To validate the assumption that pore length is approximately half guard cell length, before running the model, we calculated the relationship between the guard cell length and the stomatal pore length. ImageJ was used to measure the length of the guard cell and corresponding pore length of 40 stomata on each of 50 adaxial images and 50 abaxial images where the stomatal pore was clearly visible. Images were from different genotypes to ensure they were representative of all 200 genotypes. This data was then used to plot a regression (R^2^ = 0.98) and estimate pore length as 0.543 of the guard cell length (Supplementary Information; Figure S1B). Rather than using the conventional assumption of 0.5, we used this empirically derived value (0.543) in our model.

#### 2.3.4 Analyses and Visualisation of Data

Data analysis and visualisation were performed with R (R Core Team 2021) using packages dplyr (Wickham et al. 2023), ggplot2 (Wickham 2016), gridExtra (Baptiste 2017) and tidyr (Wickham et al. 2024). We have not yet performed any contextual statistics on this data set, as it is part of a larger study and not the primary focus of our work here. Instead, we provide qualitative descriptions of observable trends, with mean values presented.

## 3 Results

In large scale field experiments including measurements of over 1,200 leaves, our FieldDino approach reduced the time taken for stomatal conductance measurements and microscopy imagery of the adaxial and abaxial surfaces to < 10 s per leaf, and < 15 s per leaf with the inclusion of chlorophyll fluorescence. Total time per measurement averaged 1 min with the inclusion of leaf selection and movement from plot to plot.

### 3.1 Stomatal Conductance and Chlorophyll Fluorescence

**Figure 3:**
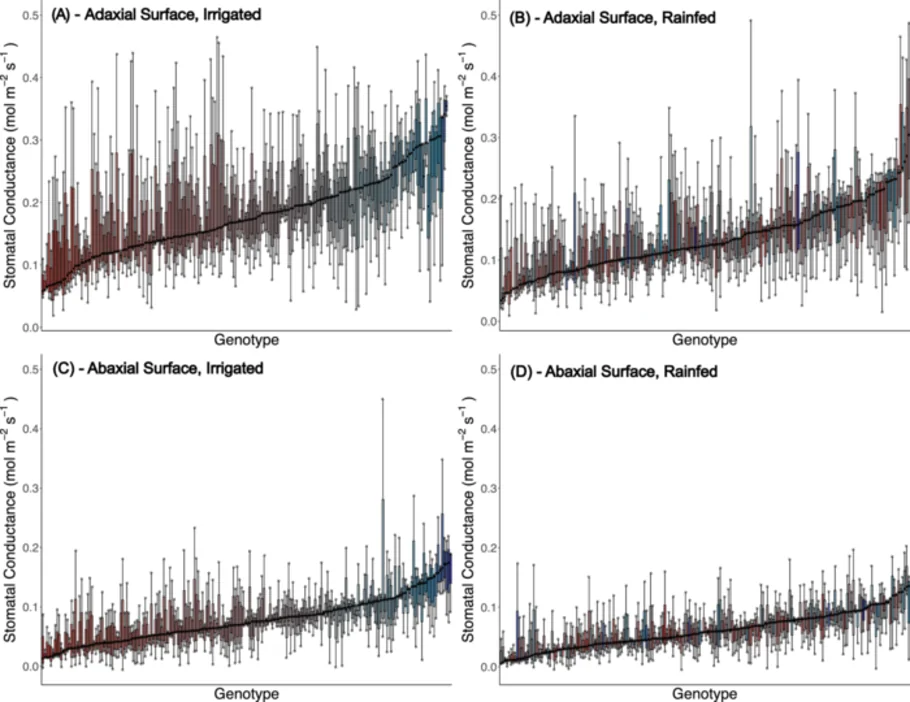
Genotypic distribution of operational stomatal conductance (*g*_*sop*_) across 200 wheat genotypes under two watering treatments. (A) Adaxial surface irrigated; (B) adaxial surface rainfed; (C) abaxial surface irrigated; and (D) abaxial surface rainfed. Genotypes are ranked by median operational stomatal conductance (*g*_*sop*_). Colour assigned to each genotype based on irrigated stomatal conductance. Thick horizontal lines within boxes indicate the median and boxes indicate the upper (75%) and lower (25%) quartiles. Whiskers indicate the ranges of the minimum and maximum values. Points indicate individual measurements. [Color figure can be viewed at wileyonlinelibrary.com]

**Figure 4:**
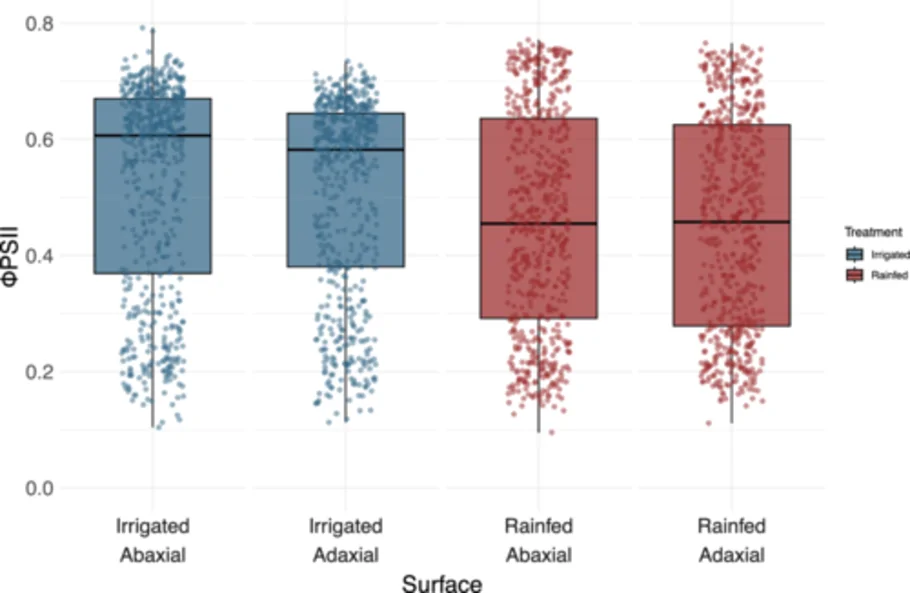
ΦPSII of the abaxial and adaxial leaf surfaces of wheat under irrigated and rainfed conditions. Thick horizontal lines within boxes indicate the median and boxes indicate the upper (75%) and lower (25%) quartiles. Whiskers indicate the ranges of the minimum and maximum values. Points indicate individual measurements. [Color figure can be viewed at wileyonlinelibrary.com]

Independent of treatment and genotype, mean adaxial *g*_sop_ was greater than the abaxial *g*_sop_ (Figure 3). *g*_sop_ was also broadly increased under irrigated conditions. The LI‐600 porometer we used was able to capture wide ranging variability across genotypes with median *g*_sop_ ranging from 0.06 mmol m^‐2^ s^‐1^ to 0.39 mmol m^‐2^ s^‐1^ and 0.03 mmol m^‐2^ s^‐1^ to 0.30 mmol m^‐2^ s^‐1^ for the adaxial surface under irrigated and rainfed treatments, respectively. The quantum yield of electron transfer at photosystem II (ΦPSII) was also variable across genotypes with median ΦPSII values of 0.58 and 0.46 for the adaxial surface under irrigated and rainfed treatments, respectively (Figure 4 and Supplementary Information; Figure S4).

### 3.2 Stomatal Anatomy

**Figure 5:**
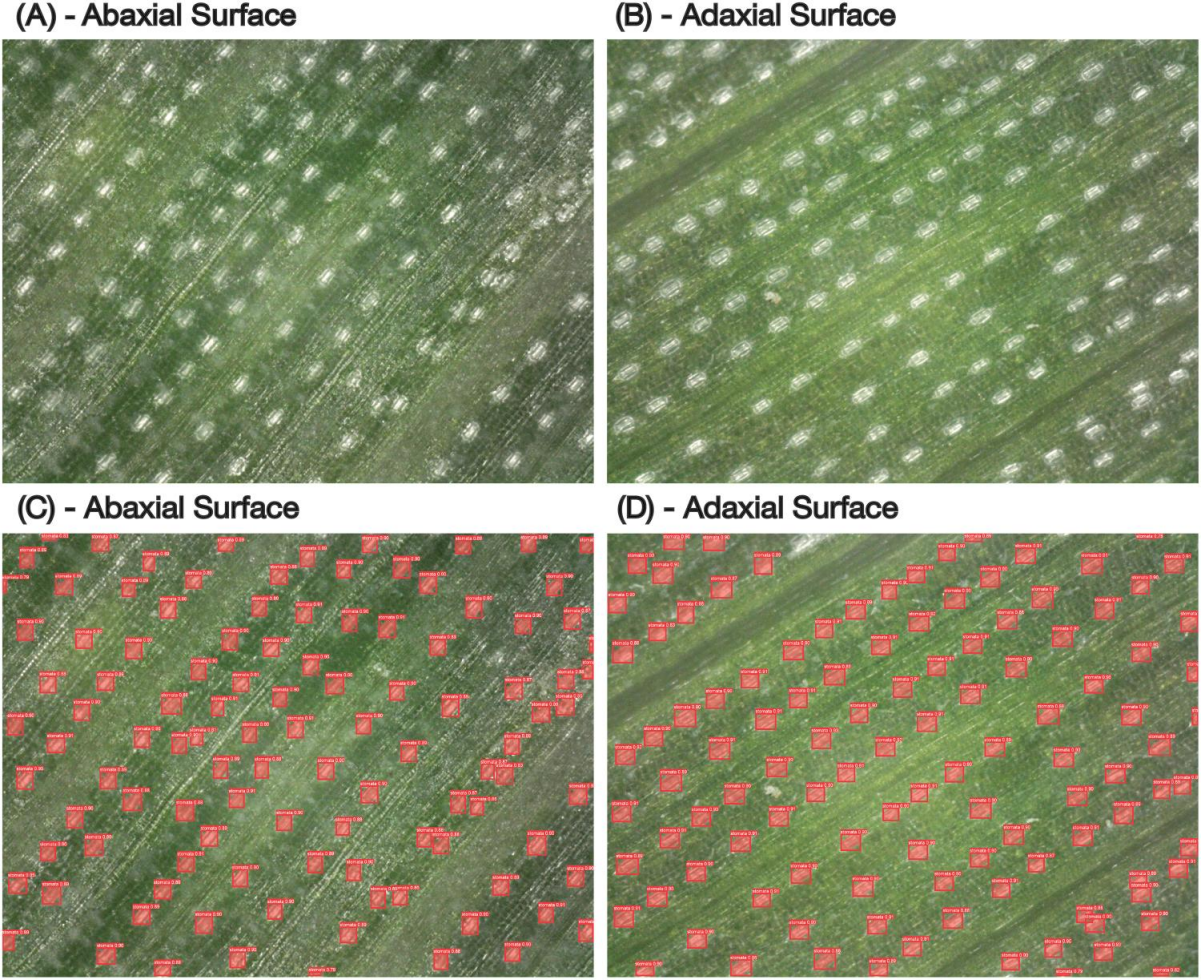
Representative images of stomatal anatomy collected *in situ* using handheld digital microscope. (A) and (C) abaxial and (B) and (D) adaxial leaf surfaces. Custom leaf clip mount was used with microscope to aid with image focussing. (A) and (B) show raw image collected using microscope, (C) and (D) show image with automatically labelled stomata using the deep learning model we trained. [Color figure can be viewed at wileyonlinelibrary.com]

**Figure 6:**
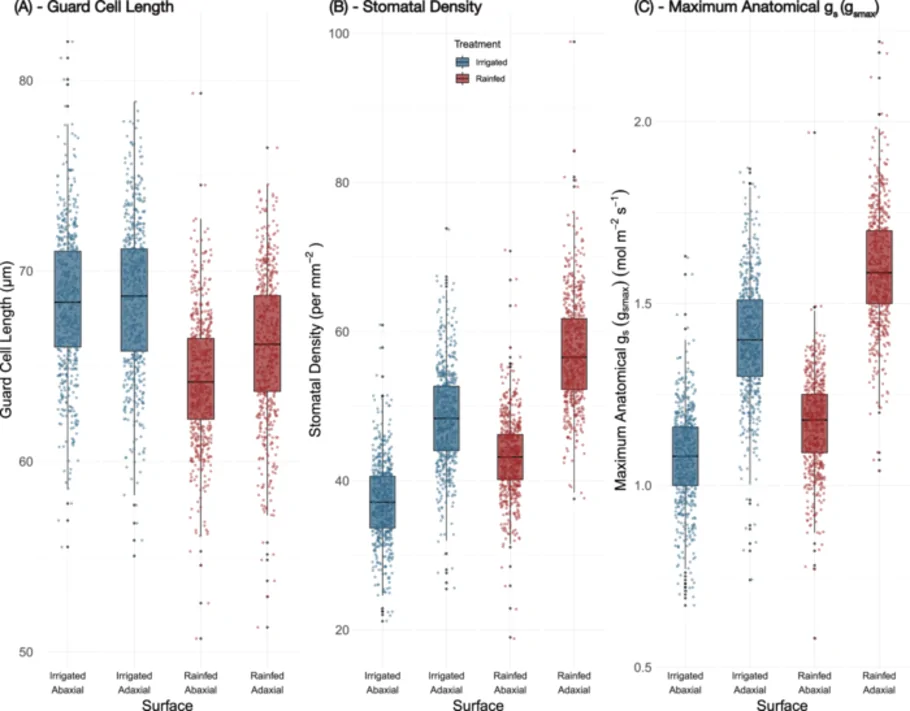
Stomatal anatomy traits of wheat under irrigated and rainfed treatments. (A) Guard cell length, (B) stomatal density and (C) maximal anatomical stomatal conductance, *g*_*smax*_. Thick horizontal lines within boxes indicate the median and boxes indicate the upper (75%) and lower (25%) quartiles. Whiskers indicate the ranges of the minimum and maximum values. Points indicate individual measurements. [Color figure can be viewed at wileyonlinelibrary.com]

**Figure 7:**
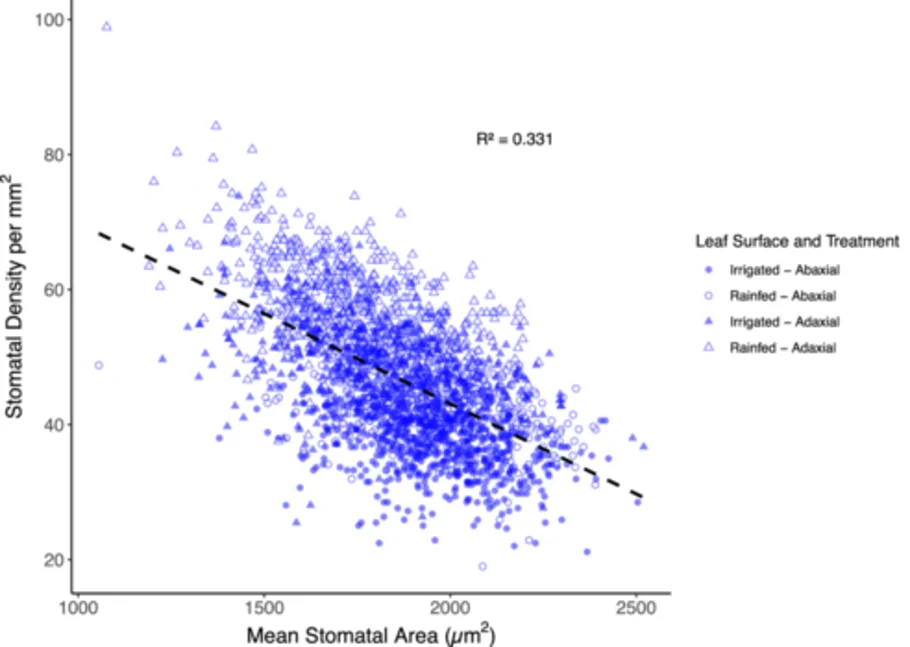
Relationship between stomatal density and stomatal area. Shaded markers represent irrigated plants and unshaded markers represent rainfed plants. Circular markers represent abaxial leaf surface and triangular represent adaxial leaf surface. [Color figure can be viewed at wileyonlinelibrary.com]

Representative microscopy images from the abaxial and adaxial leaf surfaces before and after stomatal detection using the deep learning model are shown in Figure 5. Guard cell length under rainfed conditions averaged 64.30 µm ±0.07 (abaxial) and 66.04 µm ±0.08 (adaxial), compared to 68.39 µm ±0.08 (abaxial) and 68.55 µm ±0.08 (adaxial) under irrigation (Figure 6A). Mean stomatal density was greater on the adaxial surface (52.93 ±0.141 per mm^2^) than the abaxial surface (40.25 ±0.110 per mm^2^), and under rainfed conditions compared to irrigated (Figure 6B). There was variability in stomatal density across the 200 genotypes with median stomatal densities for the adaxial surface ranging from 35.8 per mm^2^ to 62.6 per mm^2^ and 42.8 per mm^2^ to 80.7 per mm^2^ for the irrigated and rainfed treatments, respectively. Stomatal size and density were negatively correlated (R^2^ = 0.33) (Figure 7).

Stomatal anatomy was used to calculate a theoretical maximum conductance. *g*_smax_ ranged from 0.58 mol m^‐2^ s^‐1^ to 2.22 mol m^‐2^ s^‐1^ (Figure 6C). Genotypic variation in *g*_smax_ was observed with median *g*_smax_ for the adaxial surface ranging from 1.12 mol m^‐2^ s^‐1^ to 1.73 mol m^‐2^ s^‐1^ to 1.20 mol m^‐2^ s^‐1^ to 1.86 mol m^‐2^ s^‐1^ for the irrigated and rainfed treatments, respectively.

### 3.3 Combined Stomatal Physiology and Anatomy Traits

**Figure 8:**
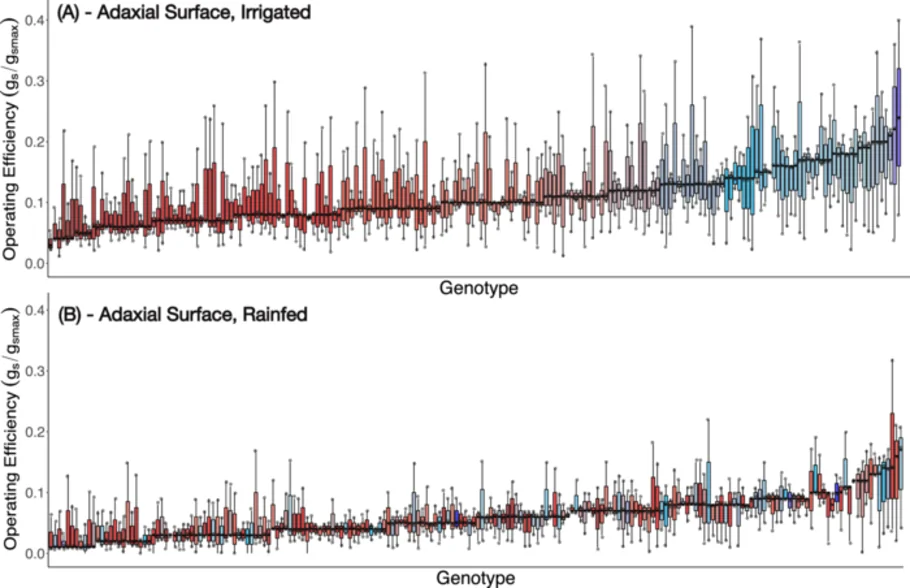
Genotypic distribution of adaxial surface stomatal conductance efficiency (*g*_*s*_ */g*_*smax*_). Data presented for 200 wheat genotypes of wheat under (A) irrigated and (B) rainfed treatments, ranked by median stomatal conductance efficiency. Colour assigned to each genotype based on irrigated stomatal conductance efficiency. Horizontal lines within boxes indicate the median and boxes indicate the upper (75%) and lower (25%) quartiles. Whiskers indicate the ranges of the minimum and maximum values. Points indicate individual measurements. [Color figure can be viewed at wileyonlinelibrary.com]

The coupled physiology‐anatomy approach we deployed allowed calculation of the stomatal conductance operating efficiency (*g*_sop_/*g*_smax_). The rapid approach provided information at scale on genotypic variation in *g*_sop_/*g*_smax_ which was observed (Figure 8) with median adaxial surface operating efficiencies of 0.03 to 0.25 and 0.01 to 0.17 for the irrigated and rainfed treatments, respectively.

## 4 Discussion

We developed a high‐throughput approach to assess stomatal physiology and anatomy in the field, applied to, and validated across large scale field trials. The approach comprises three steps, (1) a handheld porometer for in situ stomatal conductance measurements; (2) a handheld digital microscope, 3D‐printed leaf clip and data collection app for non‐destructive in situ anatomical image capture; and (3) a deep learning model for automated analyses of stomatal anatomy traits.

Conventional data collection approaches such as IRGAs and epidermal impressions are unsuitable for high‐throughput phenotyping studies. While epidermal impressions allow for long‐term sample storage and capture a larger leaf area, they require additional time‐consuming processing steps. Our approach achieved throughput rates exceeding 60 plants per hour under field conditions compared with < 10 per hour for IRGAs (Taylor and Long 2017; Salter et al. 2018). This has been achievable by developments in porometry and portable microscopy with each sample taking < 15 s for porometery and < 10 s for microscopy, while their portability enabled fast movement from plot to plot in the field. However, careful placement of the handheld microscope is essential to ensure representative sampling of the leaf surface given they capture a smaller area than epidermal impressions. These instruments offer a non‐destructive way to assess stomatal physiology and anatomy at higher throughput and lower cost than conventional techniques.

The deep learning scripts we developed to analyse stomatal microscopy images were critical in achieving high throughput, supported by the conclusions of work employing similar approaches in controlled environments (Pathoumthong et al. 2023; Sun et al. 2023; Wang et al. 2024b). Our approach extends this capability from glasshouse conditions to large scale field trials, a much needed step to ensure traits are captured under realistic environmental conditions for crop improvement (Gibbs and Burgess 2024). Whilst most deep learning model architectures are open‐access, these models often face challenges in terms of generalisability, particularly when applied across varying environmental conditions or genetic backgrounds. Given the rapid pace of model development in plant phenomics, the method we present is intentionally ‘model‐agnostic’ and compatible with any deep learning architecture. FieldDino enables the collection of reproducible microscopy images in the field, minimising variability caused by factors including outside lighting, plant growth conditions or differences in germplasm. This reduces the need for model generalisability or extensive retraining for imagery collected under different conditions, improving their applicability across different datasets.

The accuracy of contemporary porometers, such as the LI‐COR LI‐600, is evident in the similarity of our data to reported values collected with gas exchange systems. The reduced *g*_*s*_ we observed under rainfed conditions (24.4% abaxial; 28.2% adaxial) align with previously reported findings in the range of 26%–50% under mild to moderate drought (Sikder et al. 2016; Zhao et al. 2020; Chen et al. 2022). The mean stomatal length of 66.8 µm, mean width of 40.2 µm and mean stomatal density of 46.6 per mm^2^ we report are also consistent with previous research (Shahinnia et al. 2016; Wang et al. 2016; Faralli et al. 2024); as are the increases in stomatal density under rainfed conditions (Baloch et al. 2013; Wang et al. 2016; Ouyang et al. 2017) and the negative relationship between stomatal size and density (Dillen et al. 2008; Doheny‐Adams et al. 2012; Haworth et al. 2023).

By integrating measurements of both stomatal physiology and anatomy, we found reduced operating efficiency under rainfed conditions, a notable finding given the limited research investigating stomatal conductance and anatomy of the same leaves, let alone in the field (Baloch et al. 2013; Ouyang et al. 2017; Guizani et al. 2023). Each component of our approach has been used independently in wheat: a porometer for conductance data (Sharma et al. 2014; Ramya et al. 2021; McAusland et al. 2023), a handheld microscope for anatomical imaging and a deep learning model for analysis of stomatal anatomy (Sun et al. 2021, 2023; Pathoumthong et al. 2023). The novelty of our approach lies in its end‐to‐end deployment for high resolution, field‐acquired microscopy images via custom‐designed software, enabling efficient and reproducible extraction of stomatal data under field conditions. This streamlined pipeline of combined physiology and anatomy data collection has not been demonstrated in the field by other approaches, enabling simultaneous screening of multiple stomatal traits across diverse populations in large‐scale field trials, directly supporting breeding for climate resilience.

### 4.1 Limitations & Future Directions

Several limitations warrant consideration for further optimisation of this method. We used a 200x magnification microscope which captured a large leaf area. Despite high resolution, occasionally the pore was obscured, preventing direct measurements of stomatal aperture size. Instead, aperture measurements were based on established relationships between guard cell length, guard cell width and pore length (Franks and Farquhar 2007; Franks and Beerling 2009). Future work should consider 400x magnification, especially for wheat and species with smaller stomata (Pathoumthong et al. 2023). Adapting the method for species with dense epidermal hairs may require further refinement to ensure clear imaging and accurate stomatal detection. Some limitations with the microscope and porometer also remain. Although our leaf clip microscope mount and capture app aided with manual focusing, autofocus microscopes would improve image consistency further, but currently available options have limited resolution and magnification. The LI‐600 porometer lacks a proper light source, limiting its functionality for measuring chlorophyll fluorescence, particularly under changeable light conditions (Durand et al. 2022).

Assessing dynamic stomatal behaviour in the field remains underexplored. Accelerating stomatal responses could optimise coordination between carbon assimilation and water use (Lawson and Blatt 2014). Development of robust phenotyping methods that assess stomatal responsiveness at scale are required to “complete the picture” to enable integration of stomatal traits into plant breeding programs. Our approach still relies on scientists walking plot‐to‐plot to collect measurements at the leaf scale. A truly high‐throughput approach would enable reliable, remote prediction of these traits across whole field trials. Thermography and hyperspectral imaging present opportunities in this area (Deery et al. 2019; Sobejano‐Paz et al. 2020), however, would need to be validated/ground‐truthed at scales known to already work. Thus, our stomatal phenotyping approach could be viewed as a high‐throughput in‐field validation tool for emerging remote sensing techniques.

## 5 Conclusions

We have developed and validated a robust, non‐destructive high‐throughput phenotyping approach for in situ coupled stomatal physiology‐anatomy screening. This approach enables rapid assessment of stomatal density, size and anatomical maximum conductance which, alongside the measured operating stomatal conductance provides insights into stomatal operating efficiency. Our approach uses ‘off‐the‐shelf’ products, 3D‐printed parts and open‐source software, offering versatility for adaptation to assess stomatal traits of other species using a similar setup and analysis pipeline. This is a step towards accelerating our understanding of stomatal biology within the context of yield potential and yield stability under a changing climate.

## Author Contributions

William Salter: conceptualisation, Edward Chaplin and William Salter: data curation, Edward Chaplin, Guy Coleman and William Salter: formal analysis, William Salter: funding acquisition, Edward Chaplin: investigation, Edward Chaplin and William Salter: methodology, William Salter: project administration, William Salter: resources, Guy Coleman and William Salter: software, Andrew Merchant and William Salter: supervision, Edward Chaplin and William Salter: validation, Edward Chaplin: visualisation, Edward Chaplin: writing (original draft), Edward Chaplin, Guy Coleman, Andrew Merchant and William Salter: writing – review and editing.

## Conflicts of Interest

The authors declare no conflicts of interest.

## Supporting information

The following appendices are provided within the Supporting Information:
The following Supporting Figures are provided within the Supporting Information:

**Figure S1**. Approach to stomatal annotation and regression of guard cell length to pore length relationship.

**Figure S2**. Results obtained from the testing and validation process during training of the YOLOv8 M model.

**Figure S3**. Ellipse fitting for measurement of labelled stomata.

**Figure S4**. Genotypic distribution of ΦPSII across 200 genotypes of wheat under two watering treatments.

**Appendix 1**. **Methods: Germplasm** – Detailed information on the germplasm used in the field trials for validation of the method.

**Appendix 2**. **Methods: Data Analysis; Image Annotation** – Detailed information on the methods used for image annotation for model development.

## Data Availability

The data that support the findings of this study are openly available in Roboflow at https://universe.roboflow.com/narrabri-plant-physiology-hclvi/fielddino-training-set-200x. As outlined, all files for the 3D printed leaf clip, the Python script for stomatal annotation and the files and instructions for installing and using the FieldDino App are provided in a public GitHub repository which guides users through each step – https://github.com/williamtsalter/FieldDinoMicroscopy. Validation datasets for the method are available on Roboflow – https://universe.roboflow.com/narrabri-plant-physiology-hclvi/fielddino-training-set-200x.
