# Supplementary material for: FieldDino: Rapid In‐Field Stomatal Anatomy and Physiology Phenotyping
Source: Plant Cell Environ. 2025 May 27;49(10):7714–26. doi: 10.1111/pce.15639 (PMC13551662; doi:10.1111/pce.15639)
Supplement: Supplementary file 1 [file PCE-49-7714-s001.pdf]

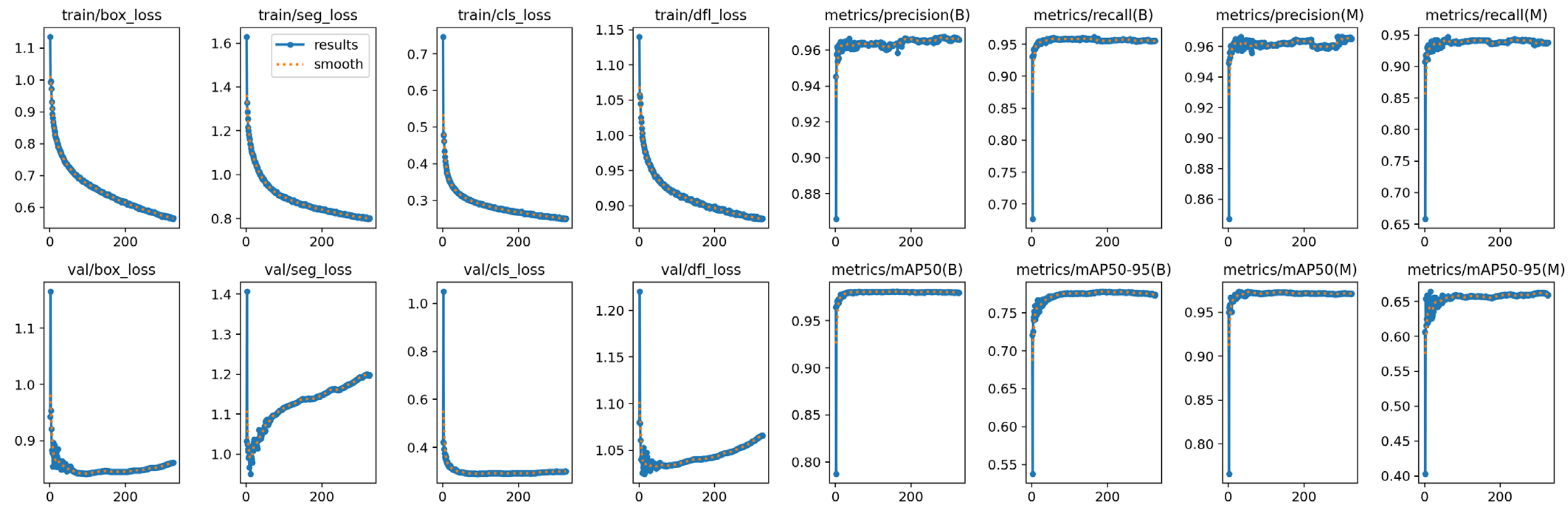
train/box_loss
train/seg_loss
train/cls_loss
train/dfl_loss
metrics/precision(B)
metrics/recall(B)
metrics/precision(M)
metrics/recall(M)
results
smooth
val/box_loss
val/seg_loss
val/cls_loss
val/dfl_loss
metrics/mAP50(B)
metrics/mAP50-95(B)
metrics/mAP50(M)
metrics/mAP50-95(M)
