# Supplementary material for: FieldDino: Rapid In‐Field Stomatal Anatomy and Physiology Phenotyping
Source: Plant Cell Environ. 2025 May 27;49(10):7714–26. doi: 10.1111/pce.15639 (PMC13551662; doi:10.1111/pce.15639)
Supplement: Supplementary file 2 [file PCE-49-7714-s005.pdf]

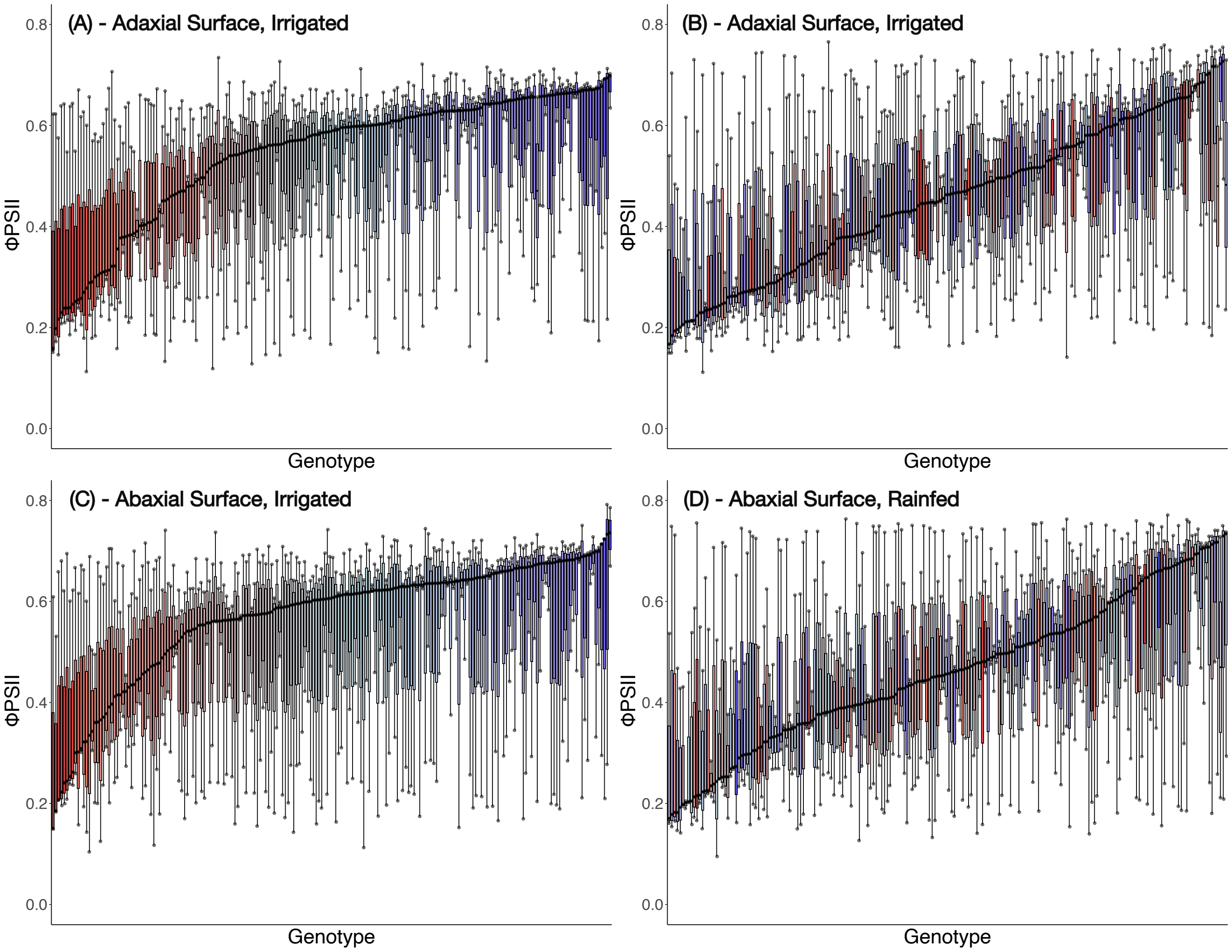

(A) - Adaxial Surface, Irrigated
(B) - Adaxial Surface, Irrigated
(C) - Abaxial Surface, Irrigated
(D) - Abaxial Surface, Rainfed
ΦPSII
Genotype
0.0
0.2
0.4
0.6
0.8
