# Supplementary material for: FieldDino: Rapid In‐Field Stomatal Anatomy and Physiology Phenotyping
Source: Plant Cell Environ. 2025 May 27;49(10):7714–26. doi: 10.1111/pce.15639 (PMC13551662; doi:10.1111/pce.15639)
Supplement: Supplementary file 3 [file PCE-49-7714-s006.pdf]

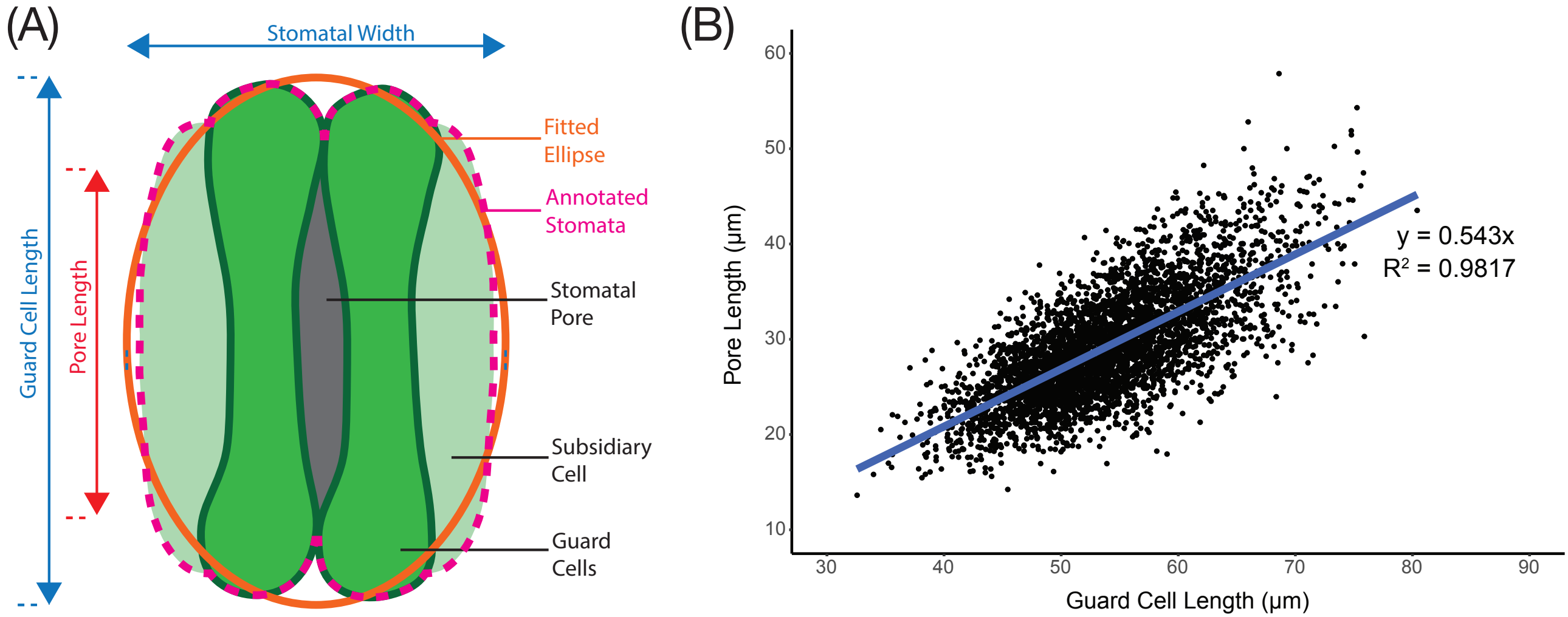
(A)
Stomatal Width
Guard Cell Length
Pore Length
Fitted Ellipse
Annotated Stomata
Stomatal Pore
Subsidiary Cell
Guard Cells
(B)
Pore Length (μm)
Guard Cell Length (μm)
y = 0.543x
R² = 0.9817
10
20
30
40
50
60
30
40
50
60
70
80
90
