# Supplementary material for: FieldDino: Rapid In‐Field Stomatal Anatomy and Physiology Phenotyping
Source: Plant Cell Environ. 2025 May 27;49(10):7714–26. doi: 10.1111/pce.15639 (PMC13551662; doi:10.1111/pce.15639)
Supplement: Supplementary file 4 [file PCE-49-7714-s003.docx]

# Supplementary Information

# Appendix 1

## Materials & Methods

#### Plant Material

The diverse germplasm comprised CIMMYT (International Maize and Wheat Improvement Center) and ICARDA (International Center for Agricultural Research in the Dry Areas) heat tolerant materials imported through the CIMMYT Australia ICARDA Germplasm Evaluation (CAIGE) program. Lines include materials from SATYN (Stress Adapted Trait Yield Nursery), EDPIE (Elite Diversity International Experiment), ESWYT (Elite Selection Wheat Yield Trial), SAWYT (Semi-Arid Wheat Yield Trial), HTWYT (High Temperature Wheat Yield Trials) and the University of Sydney including recombinants from 3 cycles of genomic selection for heat tolerance (including progeny derived from crosses among CIMMYT and ICARDA materials). Three field replicate plots of each genotype arranged in randomised blocks were sown per treatment. Plants were sown in 12 m^2^ plots (2 × 6 m) at a planting density of 100 plants m^-2^ with five planting rows.
