# Supplementary material for: FieldDino: Rapid In‐Field Stomatal Anatomy and Physiology Phenotyping
Source: Plant Cell Environ. 2025 May 27;49(10):7714–26. doi: 10.1111/pce.15639 (PMC13551662; doi:10.1111/pce.15639)
Supplement: Supplementary file 5 [file PCE-49-7714-s004.docx]

# Appendix 2

## Materials & Methods

#### Image annotation

Polygons for instance segmentation were drawn around the perimeter of the guard cells, pore and subsidiary cells (Figure S1A). Only stomata completely within the image frame were annotated.

To maintain adequate image resolution for the detection of stomatal cell details, whilst also enabling effective training, images were tiled into a 4 x 3 grid, producing 12 smaller square sub-images of 640 x 640 from the larger image. Random augmentations were applied to these sub-images prior to export from Roboflow, including random image rotation (± 15°), random brightness (± 15%) and random Gaussian blur of between 0 and 4 pixels with spread measured as the standard deviation in the horizontal and vertical direction from a single pixel. This resulted in a total of 14,928 sub-images in the dataset, split into 13,068 sub-images for training, 1,236 sub-images for model validation and 624 sub-images without augmentation for model testing. The original images (pre-augmentation) are provided as a public dataset on Roboflow: <https://universe.roboflow.com/narrabri-plant-physiology-hclvi/fielddino-training-set-200x>.

#### Model training and validation

YOLOv8 segmentation models pretrained on the Common Objects in Context dataset were finetuned using the stomata image dataset. All available model sizes were trained, including nano (N), small (S), medium (M), large (L) and extra-large (X). Models were set to train for 1,000 epochs but would cease training if no improvement in performance was made over the previous 100 epochs. The N, S, M, L and X models were trained for 315, 115, 326, 456 and 239 epochs, respectively.

Model performance was compared with precision (P), recall (R), F1 score, mAP@0.5 and mAP@[0.5:0.95] of the mask. Computational efficiency of each algorithm was measured with the Floating Point Operations per Second (FLOPs) and model size, measured in number of parameters.

While accuracy measures how often a model correctly predicts the outcome, precision measures how often the positive class is correctly predicted by the model and thus, how often positive predictions by the model are correct. Recall determines the ability of a model to find all stomata in an image, therefore acting as a measure of whether the model can find all instances of the stomata class. The relationship between the precision and recall metrics is provided by the F1 value (harmonic mean of precision and recall) with the ways in which precision, recall and F1 score are calculated summarised below (Casado-García *et al.*, 2020; Padilla *et al.*, 2021; Hicks *et al.*, 2022; Paniego *et al.*, 2022; Pathoumthong *et al.*, 2023; Wang *et al.*, 2024*b*).

Precision is defined as:

$$Precision=\frac{true positives}{true positive+false positives}$$

Recall is defined as:

$$Recall=\frac{True Positive Stomata}{Total Number of Stomata}$$

F1 Score is the harmonic mean of precision and recall which is defined as:

$$F1 Score=2 x \frac{Precision x Recall}{Precision+Recall}$$

Intersection over Union (IoU) provides an indication of model performance compared with the ground truth annotations, by calculating overlap between the predicted and ground truth regions. Intersection is the area of overlap, representing the region where the prediction has correctly identified the object. Union is the total area which is covered by both the predicted and ground truth segmentation masks (Lin *et al.*, 2021; Paniego *et al.*, 2022).

$$Intersection over Union (IoU)=\frac{Area of Intersection}{Area of Union}$$

For single-class models average precision (AP) presents a more comprehensive picture of model performance at different IoU thresholds, by computing the area under the precision-recall curve. The commonly used AP@0.5 reflects the AP of the stomata class when used with an IoU threshold of 0.5. A more comprehensive metric, mAP@0.5:0.95 represents the AP over a range of IoU thresholds in increments of 0.05 from 0.5 to 0.95. This therefore assesses the model’s performance at different levels of overlap between the predicted and ground truth boxes (Paniego *et al.*, 2022).

Based on these metrics, YOLOv8-M was chosen for stomatal analysis. The model achieved an mAP@0.5 of 97.1% and mAP@[0.5:0.95] of 65.9% (Figure S2).
